# Domain Organization, Catalysis and Regulation of Eukaryotic Cystathionine Beta-Synthases

**DOI:** 10.1371/journal.pone.0105290

**Published:** 2014-08-14

**Authors:** Tomas Majtan, Angel L. Pey, Roberto Fernández, José A. Fernández, Luis A. Martínez-Cruz, Jan P. Kraus

**Affiliations:** 1 Department of Pediatrics, University of Colorado, School of Medicine, Aurora, Colorado, United States of America; 2 Department of Physical Chemistry, Faculty of Sciences, University of Granada, Granada, Spain; 3 Department of Physical Chemistry, Faculty of Science and Technology, University of the Basque Country (UPV/EHU), Leioa, Spain; 4 Structural Biology Unit, CIC bioGUNE, Derio, Bizkaia, Spain; University of South Florida College of Medicine, United States of America

## Abstract

Cystathionine beta-synthase (CBS) is a key regulator of sulfur amino acid metabolism diverting homocysteine, a toxic intermediate of the methionine cycle, via the transsulfuration pathway to the biosynthesis of cysteine. Although the pathway itself is well conserved among eukaryotes, properties of eukaryotic CBS enzymes vary greatly. Here we present a side-by-side biochemical and biophysical comparison of human (hCBS), fruit fly (dCBS) and yeast (yCBS) enzymes. Preparation and characterization of the full-length and truncated enzymes, lacking the regulatory domains, suggested that eukaryotic CBS exists in one of at least two significantly different conformations impacting the enzyme’s catalytic activity, oligomeric status and regulation. Truncation of hCBS and yCBS, but not dCBS, resulted in enzyme activation and formation of dimers compared to native tetramers. The dCBS and yCBS are not regulated by the allosteric activator of hCBS, S-adenosylmethionine (AdoMet); however, they have significantly higher specific activities in the canonical as well as alternative reactions compared to hCBS. Unlike yCBS, the heme-containing dCBS and hCBS showed increased thermal stability and retention of the enzyme’s catalytic activity. The mass-spectrometry analysis and isothermal titration calorimetry showed clear presence and binding of AdoMet to yCBS and hCBS, but not dCBS. However, the role of AdoMet binding to yCBS remains unclear, unlike its role in hCBS. This study provides valuable information for understanding the complexity of the domain organization, catalytic specificity and regulation among eukaryotic CBS enzymes.

## Introduction

Methionine (Met) is an essential sulfur amino acid for mammals and its metabolism comprises two intersecting metabolic pathways: the methionine cycle, found in all tissues, and the transsulfuration pathway, which occurs in a limited number of tissues, but mainly in liver and kidney [1]. Both pathways compete for homocysteine (Hcy), a central intermediate that has been formed from Met. While in the methionine cycle Hcy is converted back to Met by either methionine synthase or betaine-homocysteine methyltransferase, in the transsulfuration pathway Hcy is irreversibly converted to cysteine (Cys). The transsulfuration pathway is believed to be the sole route for Cys synthesis in vertebrates [2]. Thus, Hcy formation and its distribution between these two pathways represents an occasion for regulatory intervention. Cystathionine beta-synthase (CBS) is the enzyme, which regulates the flux of Hcy through the transsulfuration pathway and thus commits Hcy to the synthesis of Cys [3]. Deficiency in CBS results in a serious metabolic disorder, homocystinuria, clinically manifested chiefly by connective tissue defects, mental retardation and thromboembolism [4].

**Figure 1:**
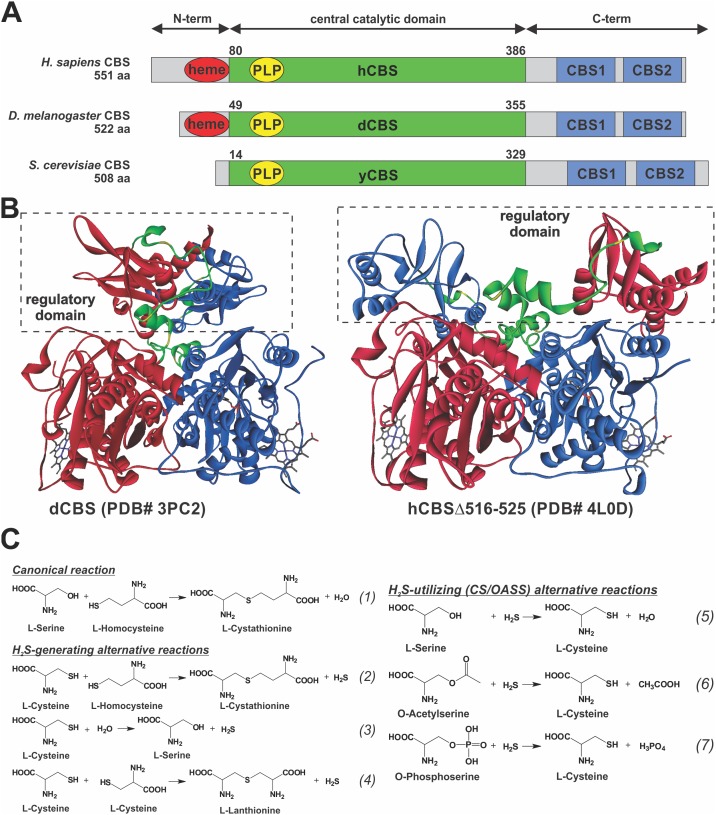
Domain architecture and structure of CBS enzymes and reactions catalyzed by CBS. (A) Domain architecture of CBS enzymes from *H. sapiens* (hCBS), *D. melanogaster* (dCBS) and *S. cerevisae* (yCBS). Regions corresponding to the central catalytic domain (green) and CBS domain (blue) as well as presence of the cofactors (heme in red, PLP in yellow) are indicated. (B) Crystal structures of dCBS (PDB #3PC2) and hCBSΔ516–525 (PDB #4L0D). Subunits within the dimers are distinguished by red or blue color, while the linker connecting the catalytic domain with the regulatory domain (dashed box) is highlighted in green. Yellow color highlights the residues in the connecting linker, which were targeted by mutagenesis (for more details see **Fig. 2**). Cofactors, heme and PLP, are shown as sticks. (C) Reactions catalyzed by CBS leading to Cth, H_2_S and Cys generation that were characterized in this study.

Considering the importance of CBS in sulfur amino acid metabolism, it is interesting that domain organization, quaternary structure and regulatory mechanism of CBS enzymes are not conserved across phyla (**Fig. 1A–B**). The extensively studied human CBS (hCBS) is a homotetrameric enzyme of 63 kDa polypeptides, each consisting of three distinct domains (reviewed in [3], [5]). The N-terminal domain of hCBS binds heme, which binds via a Cys/His ligation [6]. The origin and role of the heme in CBS is still an enigma and it is believed to function as a redox sensor [5] and/or to play a structural role facilitating a correct folding [7], [8]. The highly conserved central region forms a catalytic domain containing the PLP cofactor. The C-terminal domain houses a tandem of CBS domains, a structural motif known to bind adenosine nucleotides and to regulate protein function [9]. Indeed, the catalytic activity as well as kinetic stability of hCBS is increased upon interaction of S-adenosyl-L-methionine (AdoMet) with the CBS domains [10], [11]. In comparison to hCBS, the variability in domain organization can be illustrated on CBS species that have been experimentally documented so far [12]–[15]. The presence of heme is unique among the PLP-dependent enzymes and, moreover, not all eukaryotic CBS enzymes contain this cofactor. As an example, *Saccharomyces cerevisiae* CBS (yCBS) does not contain heme [13], while *Drosophila melanogaster* CBS (dCBS) does [14], [16]. Similarly, while majority of CBS enzymes contains the regulatory domain, only the mammalian CBS, such as hCBS, appears to be regulated by AdoMet; dCBS and yCBS are not [13], [14]. The regulatory domain seems to be essential for oligomerization as its removal from a full-length yCBS and hCBS native tetramers yields dimeric truncated enzymes [17], [18]. On the contrary, the full-length dCBS forms native homodimers [14].

In addition to the canonical condensation of Hcy with L-serine (Ser) into cystathionine (Cth) and water (**Fig. 1C**, reaction (1)), several alternative reactions have been described for the previously characterized CBS enzymes [3], [19]–[21]. A recent recognition of H_2_S as a physiologically important gaseous signaling molecule and the implication of the two transsulfuration enzymes, CBS and cystathionine gamma-lyase (CGL), in H_2_S biosynthesis [22] led to exploration of alternative H_2_S-generating reactions (**Fig. 1C**, reactions (2–4)). These reactions utilize Cys instead of Ser following either β-replacement or β-elimination mechanism to yield H_2_S. In addition, as the conserved CBS catalytic core is shared by both CBS and O-acetylserine sulfhydrase (OASS; also known as cysteine synthase (CS)) enzymes [6], [23], it was not surprising that CBS can catalyze a formation of Cys by utilizing H_2_S (**Fig. 1C**, reactions (5–7)) [19], [24]. However, physiological relevance of these alternative reactions remains to be answered.

In this study, we addressed several structural, catalytic and regulatory features of this crucial enzyme of sulfur amino acid metabolism. Side-by-side comparison of three eukaryotic CBS enzymes from yeast, fruit fly and human revealed their unique characteristics and properties. We discuss how the acquisition or loss of specific features shaped the enzyme properties and provide hypothesis explaining the complexity of domain organization, regulation and catalytic specificity among eukaryotic CBS enzymes.

## Materials and Methods

### Chemicals

Unless stated otherwise, all chemicals were purchased from Sigma or Fisher Scientific. L-[^14^C(U)]-serine was obtained from PerkinElmer Life Sciences.

### Preparation of CBS constructs

We used our established constructs for hCBS as models for cloning of yCBS and dCBS into pGEX-6P1 (GE Healthcare) and/or pET-28a (Novagen) vectors and for preparation of their C-terminally truncated forms [8], [25]. **Table S1** lists all the oligonucleotides used for subcloning and mutagenesis.

Briefly, the coding sequence of yCBS was PCR amplified from a previously prepared pGEX-5×1-yCBS plasmid [13] using the 656 and 657 oligonucleotides. The *Apa*I- and *Not*I-digested, gel-extracted PCR product was ligated into a similarly prepared pGEX-6P1 vector using T4 DNA ligase (NEB Biolabs). The dCBS coding sequence was PCR amplified from a recently reported pGEX-6P1-DMCBS plasmid [24] using the 824 and 825 oligonucleotides. The *Nco*I- and *Hind*III-digested, gel-extracted PCR product was ligated into a similarly prepared pET-28a vector using T4 DNA ligase (NEB Biolabs). Both yCBS and dCBS constructs, designated as pGEX-6P1-yCBS and pET28-C-dCBS, respectively, were transformed into *E. coli* XL1-Blue cells (Agilent) and their authenticity was confirmed by DNA sequencing. The pGEX-6P1-yCBS construct was later used as a template for PCR for recloning of full-length yCBS WT and truncated yCBS L345* into a pET-28a vector using the 794 and 795 and 794 and 796 oligonucleotides, respectively. The *Nco*I- and *Xho*I-digested, gel-extracted PCR product was ligated into a similarly prepared pET-28a vector using T4 DNA ligase (NEB Biolabs). Both pET28-C-yCBS WT and pET28-C-yCBS L345* constructs were transformed into *E. coli* XL1-Blue cells (Agilent) and their authenticity and the presence of a C-terminal 6xHis tag was confirmed by DNA sequencing.

**Figure 2:**
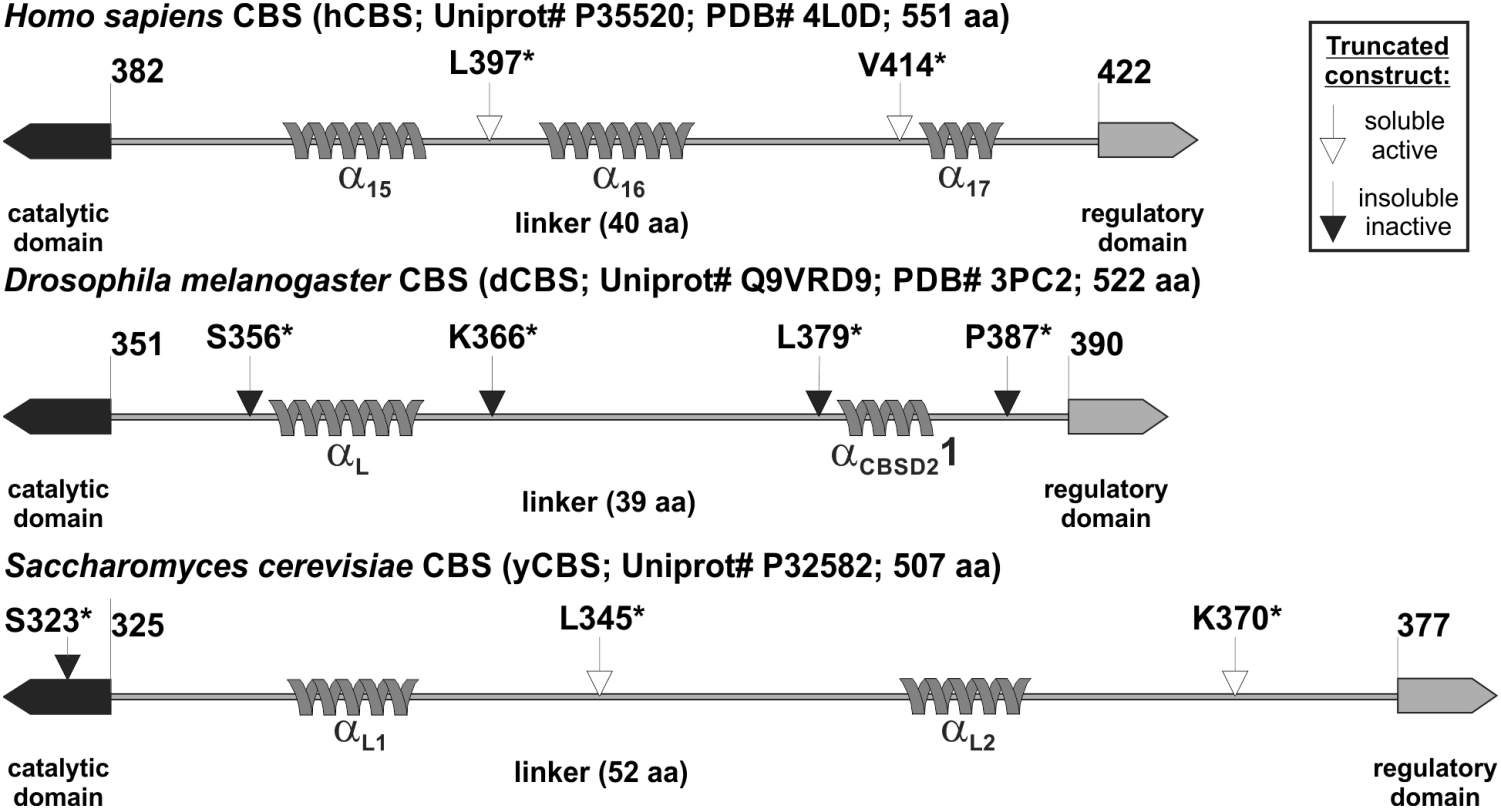
The linker connecting the catalytic and the regulatory domain in the studied CBS enzymes. Cartoon presentation of the linker sequences between the catalytic (black box pointing left) and the regulatory (grey box pointing right) domains. Grey helix shows identified (in case of hCBS – PDB #4L0D – and dCBS – PDB #3PC2) or predicted (in case of yCBS – GOR V prediction) helical secondary structures. Denomination of the linker helices in hCBS and dCBS corresponds to that used in the original reports [14], [33]. Arrows designate the introduced STOP codons, where the white arrow stands for an introduction leading to an active soluble enzyme, while black arrow designates a STOP codon introduction yielding inactive insoluble construct.

In order to prepare the various truncated forms of yCBS and dCBS lacking the regulatory domain (**Fig. 2**), the translational STOP codons were introduced using a QuikChange II XL site directed mutagenesis kit (Agilent). For yCBS, we introduced and tested three STOP codons in positions S323, L345 and K370 using the 788 and 789, 790 and 791 and 792 and 793 oligonucleotide pairs, respectively. For dCBS, we mutated four residues into STOP codons in the previously reported pGEX-6P1-DMCBS vector in positions S356, K366, L379 and P387 using the 816 and 817, 818 and 819, 820 and 821 and 822 and 823 oligonucleotide pairs, respectively. Later, the coding sequences of the truncated dCBS forms were PCR amplified using the 824 and 826, 824 and 827, 824 and 828 or 824 and 829 oligonucleotide pairs and subcloned into pET-28a vector following an analogous strategy as for the full-length form. The presence of the desired STOP codons was confirmed by DNA sequencing. The verified plasmids for full-length and truncated forms of yCBS and dCBS were finally transformed into *E. coli* Rosetta2 (DE3) expression host cells (Novagen).

### Expression and purification

Bacterial growth and protein expression were carried out at 30°C and together with preparation of soluble and insoluble crude extracts followed the previously published procedure [8], [25].

The purification of yCBS, yCBS L345* and dCBS carrying a permanent 6xHis affinity tag at the C-terminus followed the procedure recently described for human CBS variants with a few modifications [25]. After the first immobilized metal affinity chromatography step (TALON column; Clontech) and subsequent desalting on Sephadex G-25 (GE Healthcare) column, the sample was loaded onto a DEAE Sepharose (GE Healthcare) column equilibrated in the DEAE loading buffer (15 mM potassium phosphate, pH 7.2, 1 mM EDTA, 1 mM DTT, 10% ethylene glycol). The bound yCBS or yCBS L345* was washed with 2 column volumes of the DEAE loading buffer followed by 5 column volumes of the DEAE wash buffer (50 mM potassium phosphate pH 7.2, 1 mM EDTA, 1 mM DTT, 10% ethylene glycol). The enzymes were then eluted with 150 mM potassium phosphate in the DEAE loading/wash buffer. The bound dCBS was washed with 5 column volumes of DEAE loading/wash buffer (15 mM potassium phosphate, pH 7.2, 1 mM EDTA, 1 mM DTT, 10% ethylene glycol) and eluted with 50 mM potassium phosphate in the DEAE loading/wash buffer. The enzymes were buffer exchanged into the final storage buffer (20 mM HEPES pH 7.4, 1 mM TCEP) on a Sephadex G-25 column and subsequently concentrated using an ultrafiltration device (Amicon) equipped with an YM-30 (Millipore) membrane. Finally, the enzymes were aliquoted, flash-frozen in liquid nitrogen and stored at –80°C.

### Protein gel electrophoresis and Western blot analysis

Protein concentrations were determined by the Bradford method (Thermo Pierce) using bovine serum albumin (BSA) as a standard according to the manufacturer’s recommendations. Denatured proteins were separated by SDS-PAGE using a 9% separating gel with a 4% stacking gel. Native samples were separated in 4–15% polyacrylamide gradient precast gels (Mini-PROTEAN TGX, Bio-Rad). For visualization, the denatured gels were stained with Simple Blue (Invitrogen). Western blot analysis of crude cell lysates under denaturing or native conditions was performed as described previously [8].

### CBS activity measurements

The CBS activity in the classical reaction was determined by a previously described radioisotope assay using [^14^C(U)] L-serine as the labeled substrate [26]. Briefly, a purified enzyme (420 ng) was assayed in a 100 µL reaction for 30 min at 37°C. The reaction mixture contained 100 mM Tris–HCl pH 8.6, 10 mM L-serine, 0.2 mM PLP, 0.3 µCi L-[14C(U)]-serine and 0.5 mg/ml BSA. The reaction was performed in the presence or absence of AdoMet in a final concentration of 0.3 mM. The reaction mixture with enzyme was incubated at 37°C for 5 min and the reaction was initiated by addition of L-homocysteine to a final concentration of 10 mM. The reaction was terminated by an immediate cooling of the mixture in ice water and the labeled product was separated from the substrates by paper chromatography. Spots corresponding to Cth were cut-out and radioactivity was determined by using a scintillation counter.

The thermal pre-treatment of the enzyme prior the CBS activity assay was performed as described before [25]. Briefly, the purified enzyme was diluted to a final concentration of 0.1 mg/ml in Tris-buffered saline pH 8.6, 100 µM PLP. For isothermal incubation, the enzyme solutions (4×50 µl) were incubated at 37°C in 200-µl thin-walled PCR tubes in a Mastercycler gradient PCR thermal cycler (Eppendorf) for up to 96 hours. For gradual thermal denaturation, the enzyme solutions (4×50 µl) were heated in 200-µl tubes in a PCR thermal cycler from 37°C to 60°C in 0.5°C-increments with a 1 min incubation at each temperature. Aliquots (20 µl) were collected into separate tubes at designated times or temperatures and assayed for the CBS activity as described above.

The activities in the H_2_S-generating alternative reactions were measured using a colorimetric determination of H_2_S described earlier (methylene blue method [27]) with the following modifications. The reaction mixture (200 µl) contained 200 mM Tris–HCl, pH 8.6, 40 mM L-cysteine, 20 mM L-homocysteine (omitted in the cysteine β-elimination/β-replacement reaction), 0.5 mM PLP and 0.5 mg/ml BSA. The reaction was performed in the absence or presence of AdoMet in a final concentration of 0.3 mM. The mixture was incubated at 37°C for 2 min and the reaction was initiated by addition of CBS enzyme (2.5 µg) and carried at 37°C for 6 min. The reaction was terminated by a 40-fold diluting a 25 µl assay aliquot in water and mixing it with a *N, N*-dimethyl-*p*-phenylenediamine reagent and ferric chloride solution. The samples were stored in the dark at room temperature for 20 min for color development. The concentration of sulfide was determined from the absorbance at 650 nm using a standard curve prepared from sodium sulfide solutions of known concentration.

The Cys-producing activities were determined by using a colorimetric detection of generated cysteine as described earlier (ninhydrin method [28]) with the following modifications. The reaction mixture (200 µl) contained 200 mM Tris–HCl, pH 8.6, 15 mM sodium sulfide, 10 mM L-serine (or O-acetylserine or O-phosphoserine), 0.5 mM PLP and 0.5 mg/ml BSA. The reaction was performed in the absence or presence of AdoMet in a final concentration of 0.3 mM. The mixture was incubated at 37°C for 2 min and the reaction was initiated by addition of the enzyme (10 µg) and carried out at 37°C for 6 min. Reaction was terminated by removing a 50 µµl assay aliquot and mixing it with equal amounts of glacial acetic acid and acidic ninhydrin reagent. After 10 min boiling and immediate cooling, the color was stabilized by addition of denatured ethanol. The concentration of cysteine was determined from the absorbance at 560 nm using a standard curve prepared from cysteine solutions of known concentration.

One unit of activity is defined as the amount of CBS that catalyzes the formation of 1 µmol of the product in 1 hour at 37°C under above described assay conditions.

### Differential scanning calorimetry (DSC)

DSC measurements were performed in a capillary VP-DSC microcalorimeter (GE Healthcare) as described previously [11]. Briefly, samples containing 5 µM of CBS enzymes (in protein subunit) were prepared in 20 mM HEPES pH 7.4 in the presence of 50 µM PLP, and in some cases up to 400 µM AdoMet. Protein concentration was measured spectrophotometrically using the following extinction coefficients determined from acid hydrolysis: ε_280_ = 103,800 M^−1^cm^−1^ (hCBS), 79,000 M^−1^cm^−1^ (yCBS) and 119,700 M^−1^cm^−1^ (dCBS). Scans were performed in a 4–100°C range at 2–4°C/min scan rates.

Analysis of DSC transitions was performed in all cases using a two-state irreversible denaturation model [11], [29]. In this model, denaturation of the protein (or domain) is assumed to follow equation 1:
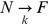
(1)Where N and F stand for the native and irreversible denatured states and *k* is the first-order rate constant. The expression used to fit the experimental DSC traces (apparent molar heat capacities, C_p(app)_, vs. Temperature) explicitly considers the experimental chemical baseline [30] as follows:



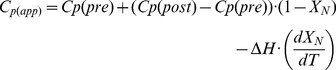
(2)

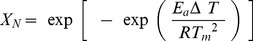
 (3)
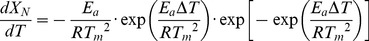
(4)Where *X*_N_ is the mole fraction of native state, Δ*H* is the denaturation enthalpy and Cp(pre) and Cp(post) are the pre- and post-transition baselines, which are considered to be a linear function of temperature, *E*_a_ stands for the activation energy and *T*_m_ for the temperature of the maximum of the transition. The two first terms in the right-hand-side of Equation 1 represent the chemical baseline and the last term describes the denaturation transition (“the peak”).

Theoretical ΔH values were evaluated using the correlation between unfolding enthalpies (at 60°) and heat capacities on the protein size (in number of residues) described by Robertson & Murphy [31]. Experimental ΔH values were determined at the experimental T_m_ of each domain/protein using the Kirchoffs equation, to compare them with the theoretical ΔH values.

### Mass spectrometry

Samples containing purified hCBS (18.4 mg/ml), yCBS (43.9 mg/ml) and dCBS (12.6 mg/ml) were examined using MALDI (matrix assisted laser desorption/ionization) mass spectrometry as described previously with a few modifications [32]. A saturated solution of CHCA (a-cyano-4-hydroxycinnamic acid) in acetonitrile/water (4:1 v:v) was used as matrix. Each sample was mixed with the matrix solution and 1 µl aliquots were deposited in the MALDI target for analysis. The AdoMet solution (10 mM AdoMet in 0.001N H_2_SO_4_, pH 3.0) and proteins were mixed with the matrix in sample:matrix (1:10 v:v) ratio and analyzed using either Synapt G2 (Waters) with a nominal resolution of 40,000 or LTQ Orbitrap XL (Thermo) with a nominal resolution of 100,000. In both cases, a minimum of 100 shots were averaged to build the final spectrum. The G2 spectrometer was calibrated before each experiment, using the peaks from polyethyleneglycol to ensure that the mass accuracy was better than 5 mDa.

### Isothermal titration calorimetry (ITC)

ITC measurements were carried out in an ITC200 microcalorimeter (GE Healthcare) essentially as described previously [11]. Briefly, protein solutions (∼20 mM in protein subunit) were prepared in 20 mM HEPES pH 7.4 and titrated at 25°C by adding 40–50 injections of 300 µM AdoMet (0.8–1 µl each). Binding thermodynamic parameters were obtained from fittings to a one- or two-independent type of sites using the software provided by the manufacturer.

## Results

### Structure of the linker between the catalytic and the regulatory domains

We analyzed and compared the primary sequence and the secondary structure elements of the region connecting the two functional domains in the studied enzymes (**Fig. 2**). The length of the linker in both heme-containing CBS enzymes is almost identical: 40 and 39 amino acid residues long in hCBS and dCBS, respectively. However, the linker in yCBS, containing only the PLP cofactor, is significantly longer containing 52 residues. The crystal structure of hCBS (PDB #4L0D) showed three helices within the linker (α_15_, α_16_ and α_17_), while the dCBS structure (PDB #3PC2) revealed only two helices (α_L_ and α_CBSD2_1) [14], [33]. By using the CDM protein secondary structure prediction server (http://gor.bb.iastate.edu/cdm/), we identified two helices in yCBS designated as α_L1_ and α_L2_. Presence of an additional helix in hCBS (α_16_) significantly differs from the unorganized central regions of the linkers in dCBS and yCBS, which may have implications for the flexibility of the linker and thus conformation of the regulatory domain towards the catalytic core as illustrated in **Fig. 1B**.

### The C-terminal truncations of dCBS and yCBS

To experimentally define the importance of the linker for a relationship between the catalytic and the regulatory domains, we introduced four and three STOPs into the sequence of dCBS and yCBS, respectively: S356*, K366*, L379* and P387* in dCBS and S323*, L345* and K370* in yCBS (**Fig. 2**). To minimize the effect of the affinity tag on the linker analysis, two types of truncated enzymes carrying either the N-terminal GST tag or the C-terminal 6xHis tag were tested.

**Figure 3:**
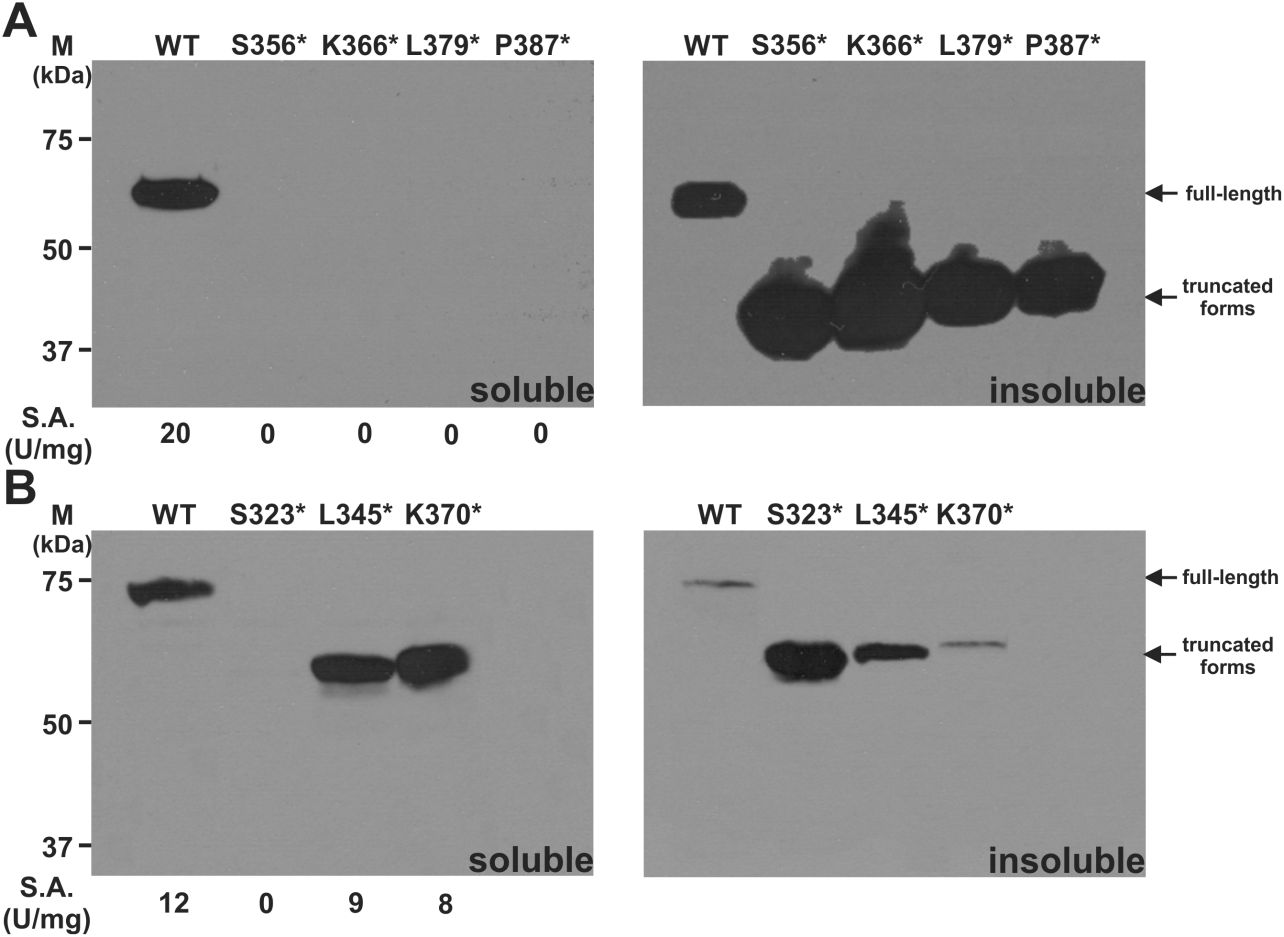
Removal of the regulatory C-terminal domain in dCBS and yCBS. Four and three STOP codons were introduced to the linker region of dCBS (A) and yCBS (B), respectively, in order to prepare the truncated enzymes. 25 ug of soluble clarified bacterial crude extract (left panels) or solubilized denatured insoluble fraction (right panels) were loaded per lane and separated on 9% SDS-PAGE gels, transferred to a PVDF membrane and probed with either monoclonal anti-6xHis antibody (ABM) (for dCBS) or monoclonal anti-GST antibody (ABM) (for yCBS). CBS specific activities are shown below the respective lanes of the soluble fractions for each construct.

As illustrated in **Fig. 3** the removal of the regulatory domain had totally different effect on dCBS compared to yCBS. None of the four tested truncated dCBS constructs yielded a soluble enzyme, which is supported by lack of CBS activity in the soluble fraction (**Fig. 3A**). As this result might likewise suggest possible problems with protein expression, we also analyzed the insoluble fractions. Strong signals from Western blot analysis after SDS-PAGE corresponding to the truncated dCBS species in insoluble fractions ruled out any problems with protein expression and similar results were obtained for the pGEX-6P1-derived constructs (data not shown). On the contrary, two out of the three tested truncated yCBS constructs (L345* and K370*) yielded active, soluble enzymes with similar results for both types of constructs: pGEX-6P1-derived GST-yCBS fusion proteins (**Fig. 3B**) as well as pET28-derived 6xHis tagged proteins (data not shown). Failure to obtain soluble active truncated enzyme for the yCBS S323* construct served as a control experiment showing that any interference within the central catalytic region (e.g. removal of the very last two residues of the catalytic domain along with the linker and regulatory domain of yCBS) yielded insoluble, inactive truncated protein.

### Initial characterization of the purified full-length and truncated CBS enzymes

**Figure 4:**
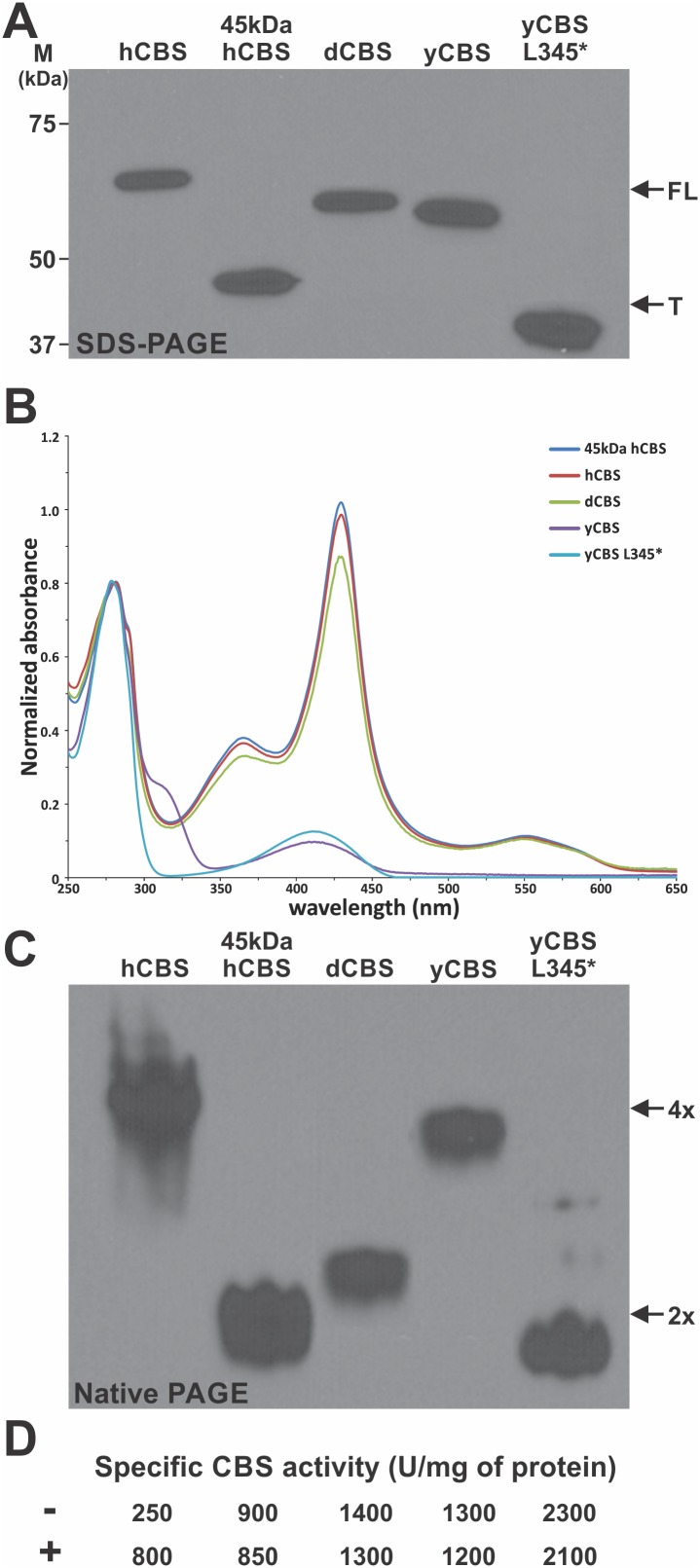
Comparison of the purified full-length and truncated CBS enzymes. (A) Homogenous purified full-length (FL; hCBS, dCBS and yCBS) and truncated (T; 45 kDa hCBS and yCBS L345*) CBS enzymes were separated on 9% polyacrylamide gel under reducing denaturing conditions (100 ng CBS per lane), transferred to a PVDF membrane and probed with monoclonal anti-6xHis antibody (ABM). (B) UV-visible spectra of purified CBSs. The enzymes were diluted in 1x Tris-buffered saline, pH 8.6 and spectra were recorded on an Agilent 8453 UV-visible spectrophotometer. (C) Purified enzymes (100 ng CBS per lane) were separated in a 4–15% polyacrylamide gel under native conditions, transferred to a PVDF membrane and probed with monoclonal anti-6xHis antibody (ABM). The 2x and 4x corresponds to native dimers and tetramers, respectively. (D) The enzymes were tested for their catalytic activity and response to the allosteric activator AdoMet in the canonical reaction. Activities are expressed as CBS specific activities (for clarity without standard deviations), where “−” designates the absence of AdoMet and “+” denotes the presence of 300 µM AdoMet.

In order to compare all three eukaryotic CBS enzymes side-by-side, we successfully purified three full-length (hCBS, dCBS and yCBS) and two truncated (45 kDa hCBS i.e. hCBS V414* and yCBS L345*) CBS enzymes to homogeneity (>95%) carrying the permanent 6xHis tag at the C-terminus (**Fig. 4A**) [25]. UV-visible spectroscopic analysis showed the presence of heme in hCBS, dCBS and 45 kDa hCBS (Soret peak at 430 nm) and its absence in yCBS and yCBS L345* (only the PLP peak at 412 nm was present) (**Fig. 4B**). Interestingly, unlike its truncated variant, the full-length yCBS showed a shoulder at around 320 nm, which may correspond to different protonation states of the PLP cofactor [34]. In case of hCBS and yCBS, removal of the regulatory domain lead to a change of the oligomeric status from tetramers to dimers as determined by native gel electrophoresis (**Fig. 4C**) and was accompanied with an increase of the catalytic activity of the truncated enzymes (**Fig. 4D**). The hCBS was the only enzyme regulated by AdoMet showing ∼3.2-fold increase in CBS specific activity upon addition of 300 µM AdoMet. Removal of the regulatory region from the AdoMet-responsive tetrameric hCBS resulted in formation of a highly active (∼3.5-fold higher activity compared to basal hCBS activity), AdoMet-unresponsive dimeric 45 kDa hCBS. Even though the basal activity of yCBS is even higher than that of 45 kDa hCBS, the truncation of yCBS yielded an almost 2-fold more active catalytic core compared to the full-length enzyme, which was also accompanied with an oligomeric status change from a native tetramer (yCBS) to a truncated dimer (yCBS L345*). Unlike hCBS and yCBS, the full-length dCBS forms native dimers, whose specific activity in the canonical CBS reaction is similar to that of yCBS.

### Catalytic variability of CBS enzymes

**Figure 5:**
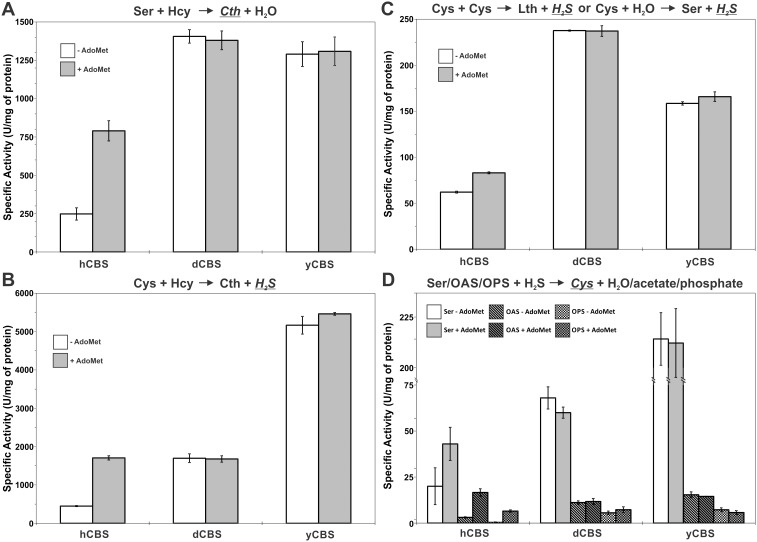
Specific activities of studied full-length CBS enzymes in various reactions. The reaction, for which the specific activities are displayed in each panel, and the detected compound (in italic and underlined) are shown above the corresponding graphs: (A) canonical reaction, (B, C) H_2_S-generating reactions and (D) Cys-producing reactions. Error bars represent standard deviations from a minimum of three independent measurements.

We assayed the purified full-length CBS enzymes and determined their specific catalytic activities in the canonical as well as alternative reactions depicted in **Fig. 1C**. Both dCBS and yCBS showed over a 5-fold higher specific activity compared to hCBS in the canonical condensation of Ser and Hcy (**Fig. 5A**). As expected, hCBS was the only studied enzyme regulated by AdoMet showing ∼3-fold increase in the presence of 300 µM AdoMet. In the alternative H_2_S-producing condensation of Cys and Hcy, all three enzymes expressed higher specific activities compared to the canonical reaction (**Fig. 5B**). While dCBS appeared to have similar activity as the AdoMet-stimulated hCBS, yCBS showed a dramatic ∼3.5-fold increase compared to the heme-containing CBS enzymes. By measuring the production of H_2_S from Cys alone, we could not differentiate between Cys desulfurase activity (β-elimination) and Cys+Cys condensation (β-replacement) (**Fig. 5C**). Again, both dCBS and yCBS showed ∼3.7x and ∼2.6x higher specific activities, respectively, compared to hCBS. Interestingly, we observed significantly lower response of hCBS to AdoMet stimulation in this reaction compared to all other tested combinations of substrates. The capability of CBS enzymes to utilize H_2_S and to form Cys was demonstrated by using either Ser as a substrate (Ser sulfhydrylase – SS – activity) or its activated forms, such as OAS and OPS (CS/OASS activity). As **Fig. 5D** shows, Ser appeared to be the only relevant substrate for CBS enzymes to form Cys. Even though CBSs are evolutionary the closest relatives to CS/OASSs, they could barely utilize the activated forms of Ser as substrates for Cys production. The yCBS showed the highest activity (∼10-fold compared to hCBS), followed by dCBS displaying ∼3.2x higher specific activity than hCBS. Taken together, both yCBS and dCBS were not regulated by AdoMet and showed higher specific activity in all tested reactions compared to hCBS.

### Thermal stability of CBS enzymes

**Figure 6:**
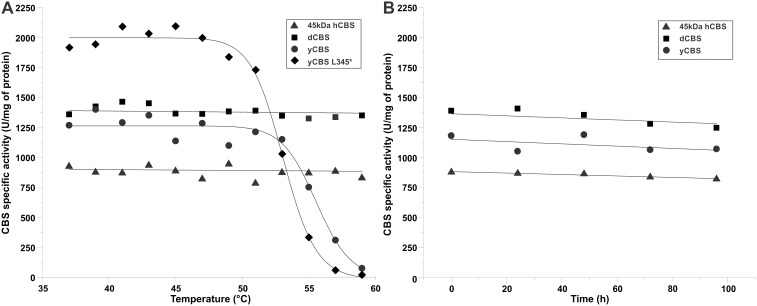
Thermal stability of the purified CBS enzymes. (A) The effect of thermal pre-treatment of full-length dCBS (squares), yCBS (circles), truncated 45 kDa hCBS (triangles) and yCBS L345* (diamonds) on their specific activities in the canonical reaction. (B) The effect of isothermal incubation (at 37°C) of the truncated 45 kDa hCBS (triangles), full-length dCBS (squares) and yCBS (circles) on their specific activities in the canonical reaction.

We have already demonstrated in our previous work, that a gradual thermal denaturation of hCBS resulted in enzyme activation due to irreversible denaturation of the regulatory domains [11], thus mimicking the stimulation by AdoMet [8], [25]. In order to assess the thermal stability of the purified CBS enzymes, we subjected dCBS and yCBS to a similar thermal pre-treatment assay (**Fig. 6A**) supplemented with an isothermal incubation at 37°C for 96 hours (**Fig. 6B**) and compared them with the hCBS. From the former analysis, it is obvious that the heme-containing CBSs (dCBS, 45 kDa hCBS) are significantly more resistant towards heat-induced denaturation and unfolding than the hemeless yCBS or its truncated form yCBS L345* (**Fig. 6A**). Clearly, such dramatic decrease in yCBS activity could not be explained by the loss of PLP as the catalytically required cofactor, present in the incubation buffer, should have efficiently compensated for its eventual loss. On the other hand, the isothermal incubation of CBS enzymes at 37°C for up to 96 hours did not show any dramatic changes (**Fig. 6B**). Altogether, our data demonstrate that, unlike yCBS, catalytic activities of the heme-containing dCBS and 45 kDa hCBS are unaffected by heating up to 60°C thus further supporting the proposed structural role of heme cofactor in CBS.

### Thermal denaturation of CBS enzymes by DSC

**Figure 7:**
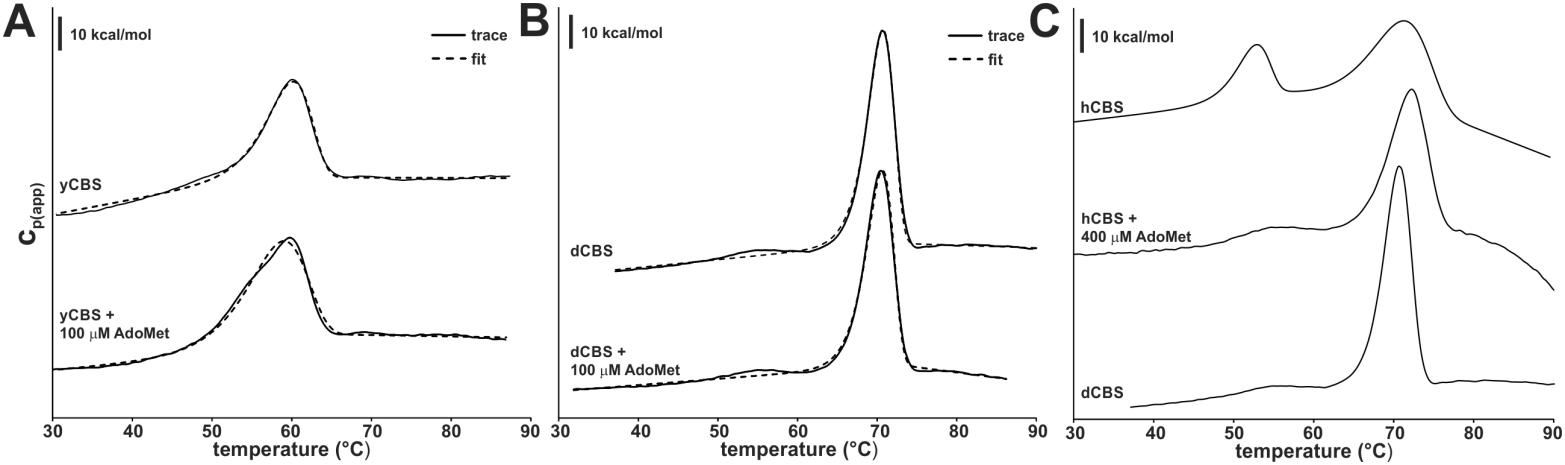
Differential scanning calorimetry (DSC) profiles of the studied CBS enzymes. DSC thermograms of yCBS (A) and dCBS (B) in the absence and the presence of 100 µM AdoMet (solid lines) overlaid with the best fit curves using a 2-state unfolding model (dashed lines). (C) Illustration of the stabilization effect of AdoMet on hCBS showing the up-shift of the first thermal transition (corresponding to the regulatory domain) by comparing this species to a largely stabilized, well-defined, sharp DSC peak of dCBS. The scan rate was 3°C/min and the protein concentration was 5 µM in protein subunit.

To study the rapid activity loss of hemeless yCBS and the resistance toward thermal denaturation of the heme-containing dCBS in more detail, we employed DSC (**Fig. 7**). As previously found for hCBS [11], denaturation of yCBS and dCBS is irreversible, strongly scan-rate dependent and described well (for the main transitions) by a simple 2-state irreversible denaturation model.

In the case of yCBS, one unfolding transition was detected with a Tm of 59.9°C and a ΔH of 158±7 kcal/mol (**Fig. 7A**). Theoretical ΔH values corresponding to denaturation of the catalytic domain alone and both the catalytic and the regulatory domains together are 227 kcal/mol and 354 kcal/mol, respectively. The unfolding of the regulatory domain (377–508) gives a theoretical value of 91 kcal/mol, and including the linker (325–508), 128 kcal/mol. Since the denaturation transition by DSC agrees well with the half-inactivation temperature (**Fig. 6A**), it is likely that this thermal transition corresponds to the irreversible denaturation of at least the catalytic domain. Therefore, based on the unfolding enthalpy we cannot say whether both domains partially unfolded or only the catalytic domain was unfolding. A kinetic analysis of yCBS denaturation provides a half-life for denaturation at 37°C of about 226 h, which is 2.2-fold lower than for the catalytic domain of hCBS [11]. The presence of 100 µM AdoMet only slightly increased the Tm about 0.2°C (**Fig. 7A**) indicating low affinity binding, low binding stoichiometry or that the ligand is not released prior to the rate limiting step of unfolding. Taken together, the data presented here suggest that yCBS is not stable when gradually heated, unfolds rapidly and AdoMet does not affect this process.

Unfolding of dCBS displays one main transition with a Tm of 70.8°C and ΔH of 388±3 kcal/mol and a second small transition with a Tm of about 55°C (**Fig. 7B**). The small transition probably reflects the unfolding of the N-terminal heme-binding domain, but it is difficult to model due to its very small signal [11]. The main transition seems to reflect the unfolding of both the regulatory and the catalytic domain, since the theoretical ΔH value of 398 kcal/mol is in excellent agreement with the experimental value. The regulatory domain of dCBS is largely stabilized reminiscent of the previously published effect of AdoMet on the regulatory domain of hCBS [11] (**Fig. 7C**). A kinetic analysis of dCBS denaturation provides a half-life for denaturation extrapolated to 37°C of about 7.4×10^7^ h, which is five orders of magnitude higher than for the catalytic domain of hCBS [11].

### MALDI-MS analysis of CBS enzymes

**Figure 8:**
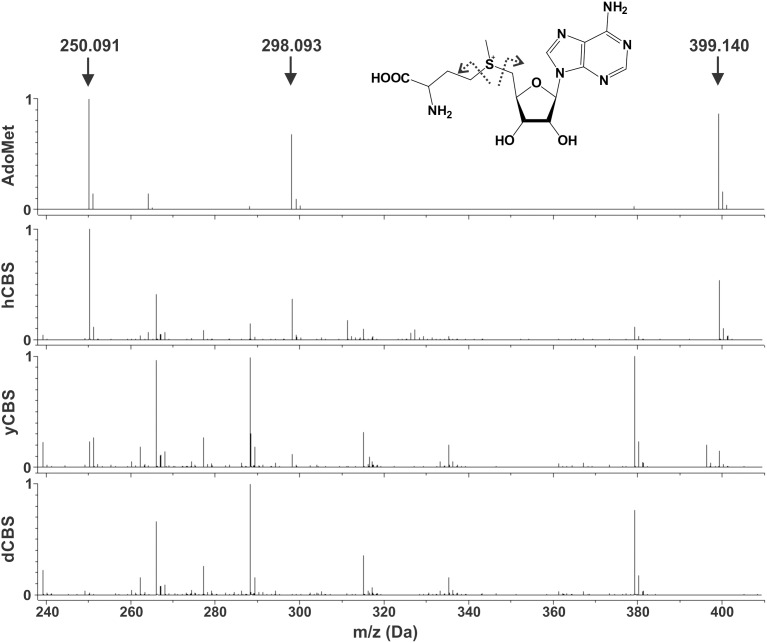
MALDI-MS analysis of full-length CBS enzymes for the presence of AdoMet. In the spectrum of authentic AdoMet, three peaks were clearly identified: 399.140 Da, 298.093 Da and 250.091 Da, which corresponds to the AdoMet protonated species and to its two protonated fragments, as indicated in the inset with dashed arrows. These exact peaks were also identified in the preparations of hCBS and yCBS, but not in the dCBS sample. To further confirm the identification, the fragmentation of the peak at 399.140 Da was also recorded (data not shown), obtaining the same fragments as for the pure substance.

The presence of only one main transition in DSC profiles of yCBS and dCBS suggested the absence of an independent transition for the regulatory domain. When compared to hCBS (**Fig. 7C**), the presence of AdoMet bound within the dCBS regulatory domain might have explained such observation. For this reason, we carried out a MALDI-MS analysis of CBS enzymes. Despite the large abundance of peaks due to the buffer, the MS spectra of hCBS and yCBS, unlike dCBS, unequivocally showed the presence of AdoMet. Peaks at positions of 399.140 Da, 298.093 Da and 250.091 Da (**Fig. 8**) correspond to the protonated parent ion and two protonated fragments. Even though the assay was not carried out in a quantitative manner, one can assume from the size of AdoMet-corresponding peaks that yCBS preparation likely contains much less bound AdoMet than hCBS, especially taking into account the relatively higher concentration of yCBS sample (see Methods section). Moreover, these amounts of AdoMet must be present in hCBS in very limited quantities, since every preparation of hCBS tested by the MS exhibited a 3-5-fold increase in the presence of AdoMet (data not shown).

Taken together, the MS analysis suggests that a significant stabilization of dCBS regulatory domain is not due to the presence of bound AdoMet within the CBS domains. Furthermore, high catalytic activity and unresponsiveness to AdoMet stimulation of yCBS as well as dCBS cannot be explained by the presence of bound AdoMet in the protein preparations.

### AdoMet binding to purified CBS enzymes by ITC

**Figure 9:**
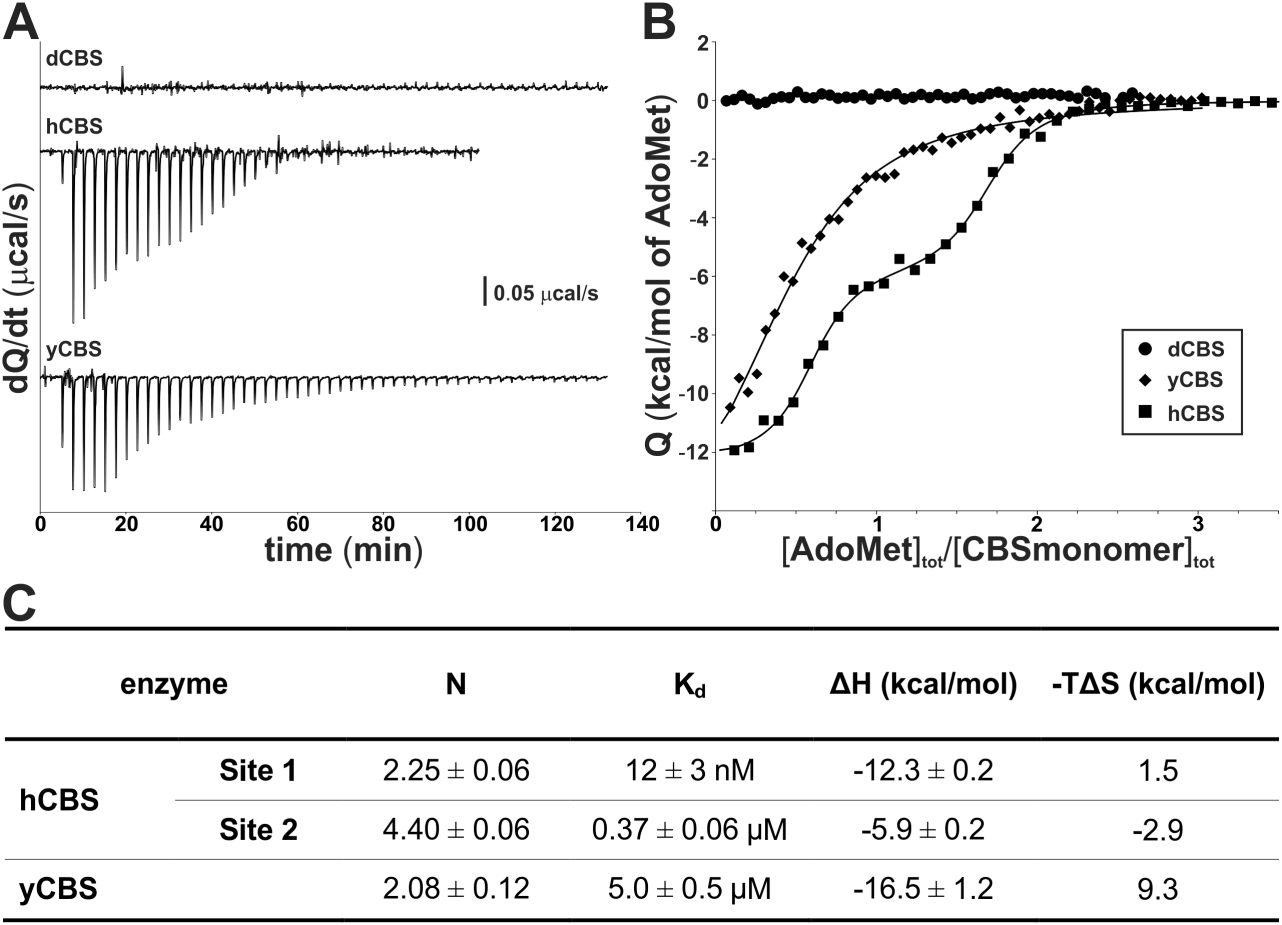
Binding of AdoMet to the examined CBS enzymes as determined by isothermal titration calorimetry (ITC). (A) Representative raw calorimetric titrations of CBS proteins (∼20 µM in protein subunit) with 300 µM AdoMet. In each experiment, 40–50 injections of 0.8–1 µl were performed. (B) Binding isotherms corresponding to full-length hCBS (squares), dCBS (circles) and yCBS (diamonds). (C) A table with thermodynamic parameters for AdoMet binding as determined from ITC measurements for yCBS and compared to the previously published data on hCBS [11]. Values of AdoMet binding sites (N) are expressed per CBS tetramer.

The MALDI-MS analysis suggested that yCBS may bind AdoMet. Therefore, we determined binding of AdoMet to yCBS and dCBS by using ITC (**Fig. 9**). Representative raw calorimetric titrations of CBS proteins with AdoMet clearly show that yCBS indeed binds AdoMet, while dCBS does not (**Fig. 9A**). However, translation of raw data into binding isotherms (**Fig. 9B**) and subsequent calculation of binding stoichiometry and thermodynamic parameters for AdoMet binding (**Fig. 9C**) showed significant differences between the values for yCBS and those previously published on hCBS [11]. Unlike hCBS, AdoMet binding to yCBS is consistent with the presence of just one type of sites with binding stoichiometry of ∼2 molecules of AdoMet per yCBS tetramer with significantly lower binding affinity (K_d_ = 5.0±0.5 µM). The lack of yCBS activation, the similar stoichiometries and binding enthalpies between the AdoMet site in yCBS and the high-affinity site in hCBS suggest those sites are alike, even though the reduction in the rate of denaturation (kinetic stabilization) exerted in yCBS by AdoMet is negligible. Taken together, yCBS binds AdoMet, but the significance of AdoMet binding in yCBS remains unclear.

## Discussion

In this study, we characterized side-by-side three CBS enzymes, from *Saccharomyces cerevisiae, Drosophila melanogaster* and *Homo sapiens.* Despite the fact that all three enzymes are from eukaryotes and have similar protein organization containing a catalytic domain and a tandem of CBS domains in the C-terminal regulatory region (**Fig. 1A**), we have shown that they significantly differ in the domain cooperativity, oligomeric status, catalysis, thermal stability, binding of AdoMet and allosteric regulation.

However, there are several exemptions to this canonical structural organization. While CBSs from parasitic protozoans *T. cruzi* and *L. major* entirely lack the regulatory domains [12], [19], *C. elegans* CBS contains a unique tandem repeat of two catalytic regions in a single polypeptide chain [15]. These examples suggest that CBS catalytic cores represent self-sustained, fully catalytically-competent independent modules. In this study, removal of the regulatory domain in hCBS and yCBS yielded highly active dimeric enzymes, while similar constructs of dCBS yielded insoluble inactive proteins. Koutmos et al. [14] suggested that the arrangement of the regulatory domain in dCBS activates the enzyme and thus is different from that of found in hCBS, where it imposes an intrasteric block [33]. Unexpectedly, removal of the CBS domains from dCBS yielded insoluble protein in spite of the fact that the crystal structure of dCBS (PDB #3PC2) does not show any significant interface or communication between the catalytic and the regulatory domains. Moreover, yCBS, which is as active as dCBS, can be further activated by a removal of the regulatory CBS domains. These contrasting results suggest that there is a communication between the catalytic core and the regulatory domain in CBS enzymes regardless of size of the shared interface or extent of physical interactions between the domains. Our results support the notion of CBS enzymes with the canonical organization adopting one of at least two different conformations e.g. as depicted in **Fig. 1B**. One conformation is similar to that of dCBS [14], where the regulatory CBS domains form a compact disk-like CBS module clearly separated from the catalytic core, as previously found in many CBS domain proteins [9], [32]. In the other conformation, represented by hCBS [33], the CBS domains physically interact with the catalytic cores and thus have a significant impact on catalytic properties of the enzyme. Indeed, we showed that removal of the regulatory domain activates both hCBS and yCBS. Moreover, similar activation of hCBS can be achieved by other means as well [17], [18]. Denaturation of the regulatory domain (e.g. by heating) [8], [35], missense mutation in the CBS domains, such as pathogenic S466L or I435T in hCBS [8], [36] or binding of the allosteric activator, such as AdoMet for the mammalian CBS enzymes [11], [37] all leads to activation of hCBS, which is most likely accompanied with a conformational change.

The relaxed substrate specificity represents the most complex property of the CBS enzymes. Comparison of kinetic parameters for the canonical Ser and Hcy condensation as well as alternative H_2_S-producing condensation of Cys and Hcy of the previously studied hCBS and yCBS suggests that, while apparent affinities of the enzymes for the substrates (K_m_) are similar, their maximal reaction velocities (V_max_) differ substantially [20], [23], [38]. The specific activities determined at saturating levels of substrates, as presented in **Fig. 5**, thus directly relate to the enzyme catalytic activity and efficiency. The dCBS and yCBS specific activities in the canonical reaction correlate nicely with the ones we reported previously [24] as well as with the V_max_ value for yCBS reported earlier [20]. As far as the alternative reactions are concerned, the insect CBS is at least as capable producing H_2_S in the studied reactions as is the AdoMet-stimulated hCBS. High catalytic activities of the investigated CBSs, mainly the yCBS, could translate *in* *vivo* into a higher flux of Hcy through the transsulfuration pathway and thus production of Cys and subsequently glutathione. Increased flux rate through the transsulfuration pathway is important for maintaining the cellular redox balance [39] and has been implicated in enhanced defense against reactive oxygen species and subsequently in increased life span of mice, fruit fly or yeast [40]–[42]. However, concurrent production of H_2_S in these alternative reactions must be tightly controlled due to its strong reducing nature, regulatory and signal transduction function at low concentrations and cellular cytotoxicity at elevated levels [22], [43], [44].

The OASS enzymes represent the closest relatives to CBS enzymes [45]; thus, it is not surprising that CBS can produce Cys by utilizing H_2_S and either Ser (SS reaction) or its activated form required by the related OASS: OAS or OPS (CS reactions). Our results suggest that Cys production from Ser could be the only relevant Cys-producing activity of CBS enzymes with yCBS being more than 5-fold more active than dCBS or hCBS (**Fig. 5D**). Clear distinction between yCBS and heme-containing dCBS and hCBS in SS reaction suggests significant differences in the active site pocket [19], [23], [24]. In support of this notion, a side-by-side comparison of CBS and CS from the protozoan parasite *Leishmania major* or *Trypanosoma cruzi* showed that their CBSs can efficiently process both Ser as well as OAS [12], [19]. In fact, catalytic efficiency of *L. major* CBS was comparable to that of CS, when using OAS and H_2_S as substrates. The significant difference in K_m_ values for both substrates suggested that both enzymes were adapted to different physiological conditions, most likely in response to various developmental stages of the parasite [19]. It is important to note, that, in addition to the N-terminal heme-binding domain, both CBS enzymes from the above mentioned parasites also lack the C-terminal regulatory domain, whose presence in the compared CBS enzymes can also affect the active site pocket geometry.

Catalytic cores of hCBS and dCBS contain the heme cofactor, which is axially coordinated by Cys/His residues in their N-terminal parts. It is unknown when and why the heme cofactor was acquired by the PLP-dependent CBS enzymes. While its function is still disputed [5], [8], [46], [47], our data presented here further support the structural role of heme in CBS. Unlike yCBS, the heme-containing dCBS and hCBS showed significantly higher resistance towards heating and thermal denaturation. The presence or absence of the regulatory domain did not play any role in thermal denaturation/stability assays as truncated yCBS or hCBS performed similarly to their full-length counterparts. We showed previously that such treatment of the full-length hCBS leads to its activation [25], which is in line with the notion of removing the intrasteric block imposed by the regulatory domain (**Fig. 1B**) [33] and supported by our recent calorimetric study [11].

**Figure 10:**
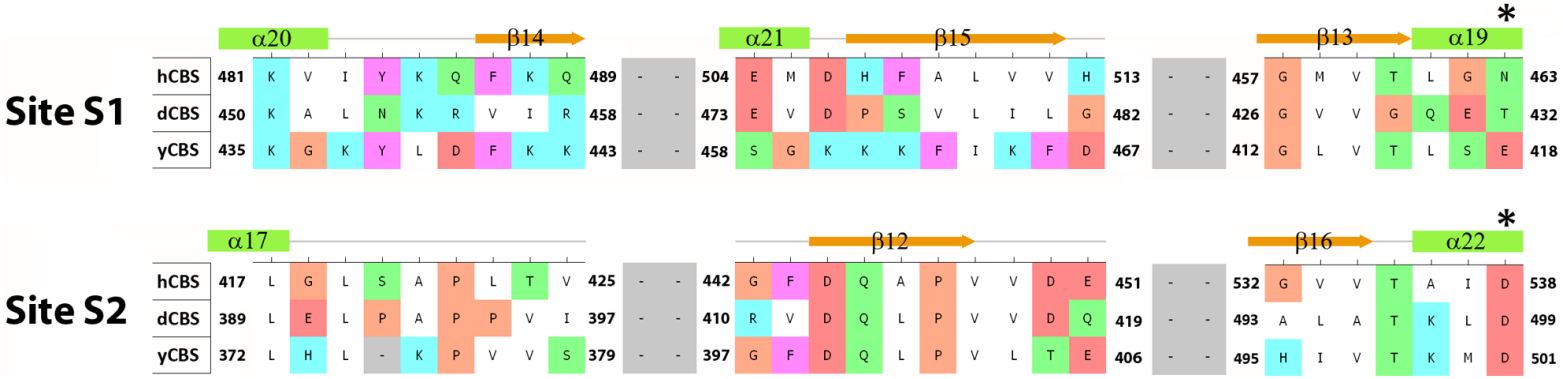
Sequence alignment of residues constituting the two potential AdoMet binding sites (S1 and S2) in hCBS, dCBS and yCBS. The residues comprising each region were extracted from a careful analysis of both the amino acid sequences and the crystal structures of dCBS and hCBS [14], [33] or 3D model of yCBS. The numbering of secondary elements shown above the sequences was adopted from the crystal structure of hCBS. The location of the conserved aspartate that most typically stabilizes the ribose ring of bound nucleotides in CBS domains is marked with an asterisk.

We showed in our previous work that the regulatory domain of hCBS has two types of AdoMet binding sites with a different stoichiometry and function (**Fig. 9C**) [11]. Here we showed that, unlike dCBS [24], yCBS and hCBS contain traces of AdoMet. An amino acid sequence alignment of the regulatory CBS domains from the three CBS enzymes (**Fig. 10**) demonstrates that mammalian and yeast enzymes share significant sequence similarity in the proposed site S2, which may be the primary binding site for AdoMet in hCBS [33]. In particular, the β16–α22 region of CBS2 domain contains a recognition motif (G*hx*S/T*xh*D/E, where *h* is a hydrophobic and *x* is any amino acid) that has been proved to favor binding of adenosine analogs in various CBS domain proteins [32]. The presence of conserved threonine and aspartate residues, the aspartate preceded by a hydrophobic amino acid, most likely favors the accommodation of the ribose and methionyl moiety of AdoMet within the site S2 of both hCBS and yCBS (**Fig. 10**). As found in other CBS domain proteins [8], [32], residues F443 (in hCBS) and F398 (in yCBS) located at the opposite side of the crevice may contribute to stabilize the adenine ring of AdoMet within the S2 cavity. The presence of a V411 residue at this position in dCBS most probably precludes binding of AdoMet at site S2 in the insect enzyme, despite the presence of conserved threonine and aspartate residues in the β16–α22 region (**Fig. 10**). Similarly, the lack of threonine/aspartate and/or a hydrophobic residue in the preceding position discards the site S1 as a suitable AdoMet binding site in all three enzymes. Nevertheless, significance of AdoMet binding in yCBS remains unclear. The absence of activation/regulation of yCBS by AdoMet (**Fig. 5**), rapid thermal denaturation (**Fig. 6** and **7**), relatively low binding affinity and stoichiometry (**Fig. 9**) contributed to the lack of kinetic stabilization as observed previously for hCBS [11]. Increased transsulfuration pathway flux mediated by the constitutively activated dCBS was found critical for adaptation of the fruit fly to its amino acid-poor diet and for the increased lifespan during dietary restriction [14], [42]. Therefore, we argue that an additional regulation of dCBS by AdoMet would be counterintuitive. Furthermore, regulation of yCBS by AdoMet would be redundant due to a concurrent presence of the transsulfuration pathway operating in the reverse direction in yeast [48], [49]. Thus the flux of sulfur through the competitive pathways can be maintained e.g. by transcriptional/translational regulation of the corresponding enzymes.

As our previous structural work suggested [33], here we have further confirmed by using series of biochemical and biophysical techniques that eukaryotic CBS with a tandem of CBS domains can adopt strikingly different conformations. While the presence of heme within the N-terminal extensions of dCBS and hCBS correlates with their increased thermal stability, the binding of AdoMet to the C-terminal regulatory domain of yCBS and hCBS does not universally activate the enzyme or kinetically stabilize its regulatory domain [11]. All three CBS enzymes showed relaxed substrate specificity catalyzing various β-replacement or β-elimination reactions. Such alternative reactivity of hCBS is integral to its proposed role as the main H_2_S generator in human body [50]. Taken together, data presented here helps our understanding of the complexity of domain organization, regulation and catalytic specificity among eukaryotic CBS enzymes.

## Supporting Information

Table S1 Oligonucleotides used in this study.
(DOCX)
