# Supplementary material for: Domain Organization, Catalysis and Regulation of Eukaryotic Cystathionine Beta-Synthases
Source: PLoS One. 2014 Aug 14;9(8):e105290. doi: 10.1371/journal.pone.0105290 (PMC4133348; doi:10.1371/journal.pone.0105290)
Supplement: Table S1 — Oligonucleotides used in this study. (DOCX) [file pone.0105290.s001.docx]

**Table S1. Oligonucleotides used in this study.**

| **Name** | **Direction** | **Sequence** | **Notes** |
| --- | --- | --- | --- |
| **656** | FWD | CTAG ***GGGCCC*** ACTAAATCTGAGCAGCAAGCC | *Apa*I site; yCBS cloning into pGEX-6P1 |
| **657** | REV | CTAG ***GCGGCCGC*** GTTATGCTAAGTAGCTCAGTAAATCC | *Not*I site; yCBS cloning into pGEX-6P1 |
| **788** | FWD | CCCAGATTCCATCAGG **TAG** TACCTAACCAAATTCG | yCBS S323 mutagenesis into a STOP codon |
| **789** | REV | CGAATTTGGTTAGGTA **CTA** CCTGATGGAATCTGGG | yCBS S323 mutagenesis into a STOP codon |
| **790** | FWD | CAATTTGTGGGATGATGACGTG **TAG** GCCCGTTTTGA | yCBS L345 mutagenesis into a STOP codon |
| **791** | REV | TCAAAACGGGC **CTA** CACGTCATCATCCCACAAATTG | yCBS L345 mutagenesis into a STOP codon |
| **792** | FWD | TGTGTTTGGTAACGCTACTGTA **TAG** GATCTTCACTTGAAAC | yCBS K370 mutagenesis into a STOP codon |
| **793** | REV | GTTTCAAGTGAAGATC **CTA** TACAGTAGCGTTACCAAACACA | yCBS K370 mutagenesis into a STOP codon |
| **794** | FWD | CTAG ***CCATGG*** GCACTAAATCTGAGCAGCAAG | *Nco*I site; yCBS WT & L345* cloning into pET-28a |
| **795** | REV | CTAG ***CTCGAG*** CACGTCATCATCCCACAAATTGTTC | *Xho*I site; yCBS L345* cloning into pET-28a |
| **796** | REV | CTAG ***CTCGAG*** TGCTAAGTAGCTCAGTAAATCCATCTTAG | *Xho*I site; yCBS WT cloning into pET-28a |
| **816** | FWD | CAACTACATGACCAAGTTCGTG **TAG** GACAACTGGAT | dCBS S356 (pGEX) mutagenesis into a STOP codon |
| **817** | REV | ATCCAGTTGTC **CTA** CACGAACTTGGTCATGTAGTTG | dCBS S356 (pGEX) mutagenesis into a STOP codon |
| **818** | FWD | GAGGCGCGCAACTTC **TAG** GAGCCGGTAAAC | dCBS K366 (pGEX) mutagenesis into a STOP codon |
| **819** | REV | GTTTACCGGCTC **CTA** GAAGTTGCGCGCCTC | dCBS K366 (pGEX) mutagenesis into a STOP codon |
| **820** | FWD | CTGGTGGTGGAGC **TAG** GCCATCGCGGAG | dCBS L379 (pGEX) mutagenesis into a STOP codon |
| **821** | REV | CTCCGCGATGGC **CTA** GCTCCACCACCAG | dCBS L379 (pGEX) mutagenesis into a STOP codon |
| **822** | FWD | CCATCGCGGAGTTGGAGCTA **TAG** GCTCCCCCGGT | dCBS P387 (pGEX) mutagenesis into a STOP codon |
| **823** | REV | ACCGGGGGAGC **CTA** TAGCTCCAACTCCGCGATGG | dCBS P387 (pGEX) mutagenesis into a STOP codon |
| **824** | FWD | CTAG ***CCATGG*** GCCCCCAACCGAAG | *Nco*I site; dCBS WT & truncates cloning into pET-28a |
| **825** | REV | CTAG ***AAGCTT*** GTGGCTGCCGCCGTTG | *Hind*III site; dCBS WT cloning into pET-28a |
| **826** | REV | CTAG ***AAGCTT*** CACGAACTTGGTCATGTAGTTGCGTATGCC | *Hind*III site; dCBS S356* cloning into pET-28a |
| **827** | REV | CTAG ***AAGCTT*** GAAGTTGCGCGCCTCCATCC | *Hind*III site; dCBS K366* cloning into pET-28a |
| **828** | REV | CTAG ***AAGCTT*** GCTCCACCACCAGTGACCGTG | *Hind*III site; dCBS L379* cloning into pET-28a |
| **829** | REV | CTAG ***AAGCTT*** TAGCTCCAACTCCGCGATGGCC | *Hind*III site; dCBS P387* cloning into pET-28a |
